# Investigation of Thermally Induced Degradation in CH_3_NH_3_PbI_3_ Perovskite Solar Cells using *In-situ* Synchrotron Radiation Analysis

**DOI:** 10.1038/s41598-017-04690-w

**Published:** 2017-07-05

**Authors:** Nam-Koo Kim, Young Hwan Min, Seokhwan Noh, Eunkyung Cho, Gitaeg Jeong, Minho Joo, Seh-Won Ahn, Jeong Soo Lee, Seongtak Kim, Kyuwook Ihm, Hyungju Ahn, Yoonmook Kang, Hae-Seok Lee, Donghwan Kim

**Affiliations:** 1 Materials & Devices Advanced Research Institute, LG Electronics, Seoul, 06763 Republic of Korea; 2 Beamline Research Division, Pohang Accelerator Laboratory, Pohang, Kyungbuk 37673 Republic of Korea; 3 Department of Materials Science and Engineering, Korea University, Seoul, Republic of Korea

## Abstract

In this study, we employ a combination of various *in-situ* surface analysis techniques to investigate the thermally induced degradation processes in MAPbI_3_ perovskite solar cells (PeSCs) as a function of temperature under air-free conditions (no moisture and oxygen). Through a comprehensive approach that combines *in-situ* grazing-incidence wide-angle X-ray diffraction (GIWAXD) and high-resolution X-ray photoelectron spectroscopy (HR-XPS) measurements, we confirm that the surface structure of MAPbI_3_ perovskite film changes to an intermediate phase and decomposes to CH_3_I, NH_3_, and PbI_2_ after both a short (20 min) exposure to heat stress at 100 °C and a long exposure (>1 hour) at 80 °C. Moreover, we observe clearly the changes in the orientation of CH_3_NH_3_^+^ organic cations with respect to the substrate in the intermediate phase, which might be linked directly to the thermal degradation processes in MAPbI_3_ perovskites. These results provide important progress towards improved understanding of the thermal degradation mechanisms in perovskite materials and will facilitate improvements in the design and fabrication of perovskite solar cells with better thermal stability.

## Introduction

Solar cells have received a significant amount of attention as an environmentally friendly and safe next-generation energy source, and, to date, solar cell active layers have been fabricated, studied, and commercialised successfully using a variety of materials and architectures. Among these, organic–inorganic hybrid perovskite solar cells (PeSCs) have come into the spotlight over the past few years owing to their cost-effective manufacturing process, flexible electronic applications, low weight, and remarkable power conversion efficiency (PCE)^1–3^. The application of organic–inorganic hybrid perovskites as light absorbers was first introduced and demonstrated by Miyasaka and co-workers in 2009^4^. Nevertheless, the PCE and stability of hybrid perovskites such as CH_3_NH_3_PbI_3_ (MAPbI_3_) and CH_3_NH_3_PbBr_3_ as sensitisers in liquid electrolyte-based dye-sensitised solar cells (DSSCs) were poor due to iodine-based redox processes. The replacement of liquid electrolytes with solid-state hole-transport layers (e.g. Spiro-OMeTAD), however, led to improvements in both stability and PCE^5–7^. As the number of researchers involved in this filed increased, the PCEs of PeSCs improved rapidly, reaching PCE of over 20% in subsequent studies as a result of innovative fabrication techniques^8–10^. In addition to achieving high PCE, solar cells must be able to function without physical and chemical degradation under various environmental conditions. Currently, however, the stability of PeSCs does not meet the PCE standards required for commercialisation^11–13^.

Typically, there are three main factors that affect the degradation of perovskites, namely air (both oxygen and moisture), UV light, and temperature (heat stress). Methylamonium (MA)-based perovskites—one of the first materials introduced into the active layer of PeSCs and also the most widely studied—have, in particular, displayed a relatively low stability to these factors^14–16^. The stability of (MA)-based perovskites has been investigated under various environmental conditions and a range of stability-enhancing techniques have been tested to date. For example, the stability to oxygen and moisture was shown to be improved somewhat by protecting the underlying perovskite film using air-stability enhancement techniques based on the introduction of inorganic or metal oxide transport layers^17, 18^ and encapsulation^19, 20^. Further, the modification of the perovskite structure from three-dimensional perovskite films to layered two-dimensional perovskite films using containing spacing layers has exhibited promising increase in stability to light soaking and humidity^20, 21^. In addition to these concerted efforts in studying air-stability enhancement techniques, the degradation mechanisms of MA-based perovskites mediated by the presence of oxygen, moisture, and UV have been researched, suggesting a route towards perovskite solar cells with long device lifetime and resistance to ambient, atmospheric, and UV light^22–27^. The degradation mechanism of MAPbI_3_ in the presence of moisture has been reported by Christians *et al*. and Leguy *et al*., who suggested the formation of a hydrate product similar to (CH_3_NH_3_)_4_PbI_6_∙2H_2_O with 3PbI_2_ under humid conditions^22, 23^. The hydrate product reversibly returns under dry conditions, but PbI_2_ limits this reversibility when left for a long time^24^. Light- and oxygen-induced degradation occurs faster than moisture-induced degradation. Xu *et al*. suggested that exposure to light (X-rays) creates dipole-aligned CH_3_-PbI_2_ defects, which lead to strain in the lattice, eventually inducing collapse of the perovskite structure into PbI_2_ and free CH_3_I and NH_3_^25^. Aristidou *et al*. and Bryant *et al*. reported that oxygen-induced degradation is initiated by the reaction of superoxide (O_2_^−^) with the MA moiety of the perovskite absorber^26, 27^. However, this degradation can be slowed down by the integration of electron extraction layers within the device architecture. In addition to the stability to air and light, thermal stability represents another key factor in the fabrication of solar cells and, currently, further improvements in thermal stability and investigations into the progress and mechanism of thermal degradation are required. In particular, the thermal reaction mechanism of perovskite materials is crucial for better understanding of their thermal stability and will facilitate improvements in both post-conditioning and synthesis of PeSCs^13, 28^. Furthermore, even though the degradation of MA into various defect states in perovskite bulk has been studied^29^, it is necessary to monitor surface degradation; this is because degradation might occur in the direction from the perovskite surface, significantly deforming not only the perovskite but also the interface between the transport layer and the perovskite absorber.

In this study, we examined the mechanism of thermal degradation in MAPbI_3_ perovskite as a function of temperature using *in-situ* surface analysis techniques. First, we fabricated well-sealed PeSCs and tested their long-term stability under various temperature and humidity conditions. While the encapsulation process was found to protect the constructed device from moisture and oxygen, in this case, thermal decomposition was not prevented and occurred from the surface top in the direction of the material bulk. The heat stress affected the PCE of the examined PeSCs, even though no changes were observable in the encapsulated MAPbI_3_ perovskite by the naked eye. In order to establish the cause of the decline in efficiency, we employed a combination of *in-situ* grazing-incidence wide-angle X-ray diffraction (GIWAXD), high-resolution X-ray photoelectron spectroscopy (HR-XPS), and near-edge X-ray absorption fine structure (NEXAFS) spectroscopy measurements to obtain information about the thermal degradation processes in the MAPbI_3_ perovskite. The comprehensive results of our *in-situ* surface analysis provided a better understanding of the important factors that need to be taken into consideration in commercial applications and the parameters affecting the thermal stability of PeSCs under different environmental conditions.

## Results and Discussion

**Figure 1:**
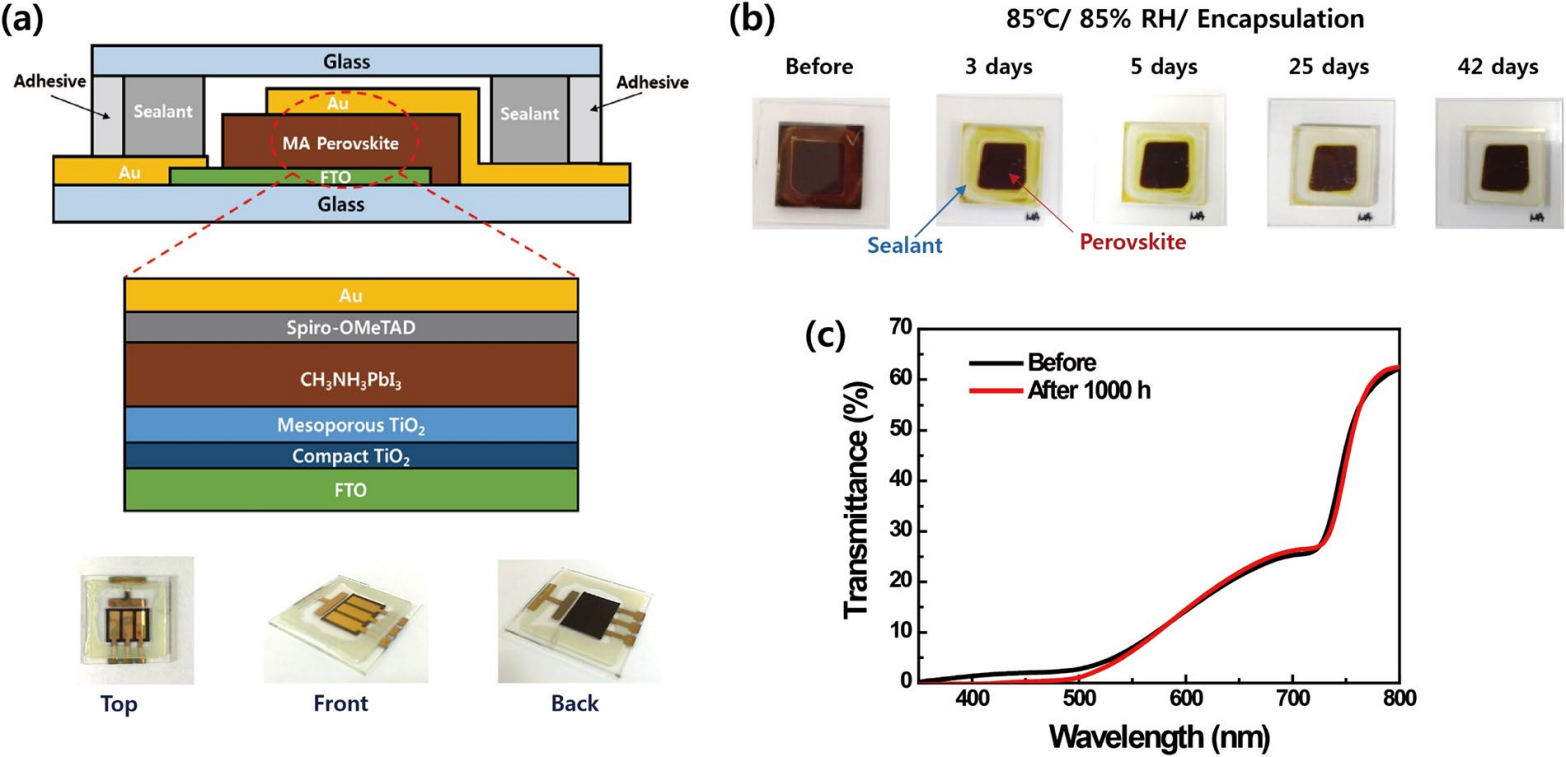
(**a**) Device architecture and images of encapsulated perovskite solar cells. (**b**) Colour of encapsulated MAPbI_3_ perovskite film observed before and after continuous heating at 85 °C and 85% relative humidity (RH). (**c**) Transmittance spectra of encapsulated MAPbI_3_ films before and after heating at 85 °C and 85% RH for 1000 h.

We employed MA-based PbI_3_ perovskite material as the light absorber in bi-layered solar cells and tested their long-term stability. The compact and mesoporous TiO_2_ layers were deposited on a fluorine-doped tin oxide (FTO) substrate as the electron selective layer. Following the deposition of MAPbI_3_ perovskite using a one-step method, a coating of a commonly employed hole-transport layer based on 2,2′,7,7′-tetrakis(*N*,*N*-di-4-methoxyphenylamino)-9,9′-spirobifluorene (Spiro-OMeTAD) was applied directly on top. Prior to testing the stability of the prepared MAPbI_3_ perovskite solar cells, we performed an encapsulation procedure under an inert argon atmosphere, as illustrated in Fig. 1a. For better encapsulation, an adhesive was applied following the sealant UV-curing procedure. Single layer MAPbI_3_ perovskite films were sealed using the same procedure as shown in Fig. 1a, and the long-term stability of encapsulated MAPbI_3_ films was tested at 85 °C and 85% relative humidity (RH). Visual analysis revealed that the aged MAPbI_3_ perovskite exhibited a black phase similar to that observed in the sample before exposure to the tested temperature and humidity conditions (Fig. 1b). Furthermore, the transmittance spectra of the encapsulated MAPbI_3_ perovskite films displayed nearly identical patterns before and after the stability test (Fig. 1c).

**Figure 2:**
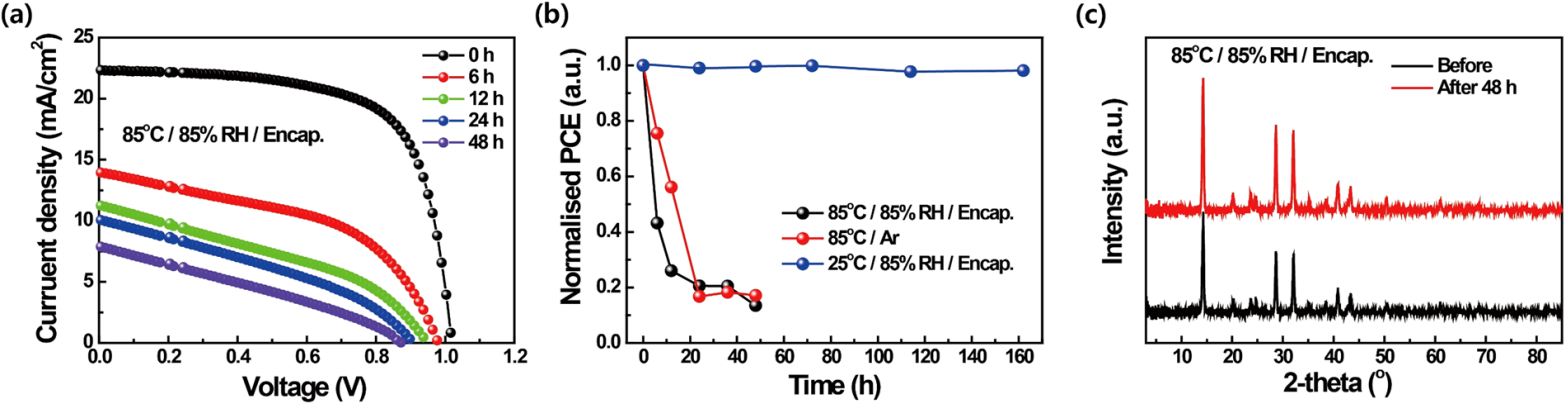
(**a**) Current–voltage curves of encapsulated MAPbI_3_ PeSCs determined as a function of exposure time at 85 °C and 85% relative humidity (RH). (**b**) Power conversion efficiencies of PeSCs examined under three different environmental conditions. (**c**) The X-ray diffraction (XRD) patterns of encapsulated MAPbI_3_ films before and after heating to 85 °C at 85% RH over 45 h.

While we observed no significant changes in the colour and transmittance spectra of the prepared films after the long-term stability test, the performance of PeSCs decreased dramatically as the stability test at 85 °C and 85% RH progressed in duration (Fig. 2a). The values of short-circuit current density (J_sc_), open-circuit voltage (V_oc_), and fill factor (FF) were obtained for the pristine PeSC from the J–V curve in the reverse scan as 22.33 mA/cm^2^, 1.02 V, and 68%, respectively, yielding a PCE of 15.45% under standard AM 1.5 conditions. However, the PCE of the PeSCs degraded significantly to about 20% of its original value (J_sc_ = 10.09 mA/cm^2^, V_oc_ = 0.91, FF = 35%, and PCE = 3.18%) after only 24 h at 85 °C and 85% RH. In order to establish the main environmental parameters affecting the performance of encapsulated PeSCs, we conducted long-term stability tests on MAPbI_3_ perovskite solar cells under three different environmental conditions for up to 160 h (Fig. 2b). In order to avoid any potential interplay between humidity and heat stress, the encapsulated device was tested under high humidity conditions (85% RH) at both room temperature (25 °C) and high temperature (85 °C). Devices prepared without encapsulation were also tested in an Ar-filled glove box at same high temperature (85 °C). At room temperature, the PCE of the encapsulated PeSCs remained stable under the high humidity conditions (25 °C and 85% RH). In contrast, the encapsulated devices exposed simultaneously to high humidity and high temperature conditions, and non-capsulated devices exposed to high temperature in the Ar-filled glove box, both exhibited poor stability. These results indicate that a well-sealed device is protected from moisture but not from thermal degradation, even at a fairly low temperature of 85 °C. Therefore, the main cause of diminished performance in well-encapsulated PeSCs is associated with heat stress. In order to confirm the changes in crystallinity as a function of environmental conditions, X-ray diffraction (XRD) patterns of encapsulated MAPbI_3_ films were collected both before and after continuous heating at 85 °C and 85% RH for 48 h. Interestingly, we did not observe any significant differences in the XRD patterns of the examined samples. Even though the bulk properties of the materials did not display any changes observable by the naked eye and both the transmittance and XRD spectra were fairly similar, we hypothesised that thermal degradation might be occurring in the direction from the perovskite surface, thereby significantly deforming the interface between the transport layer and the perovskite absorber, and, thus, reducing the PCE. In order to verify this hypothesis, we investigated in detail the changes in the surface of MAPbI_3_ perovskite taking place as a function of heat stress using *in-situ* 2D GIWAXD, HR-XPS, and NEXAFS measurements. These techniques provide information about the change of crystallinity, chemical structure, and orientation of the organic ligand (MA) in the surface, respectively. All measurements were conducted under vacuum (GIWAXD~10^−2^, HR-XPS, NEXAFS ~1.0 × 10^−10^ Torr) to ensure that air and humidity do not affect the samples^30^ and, thus, the changes observed in the surface of MAPbI_3_ arise solely as a result of heat stress.

**Figure 3:**
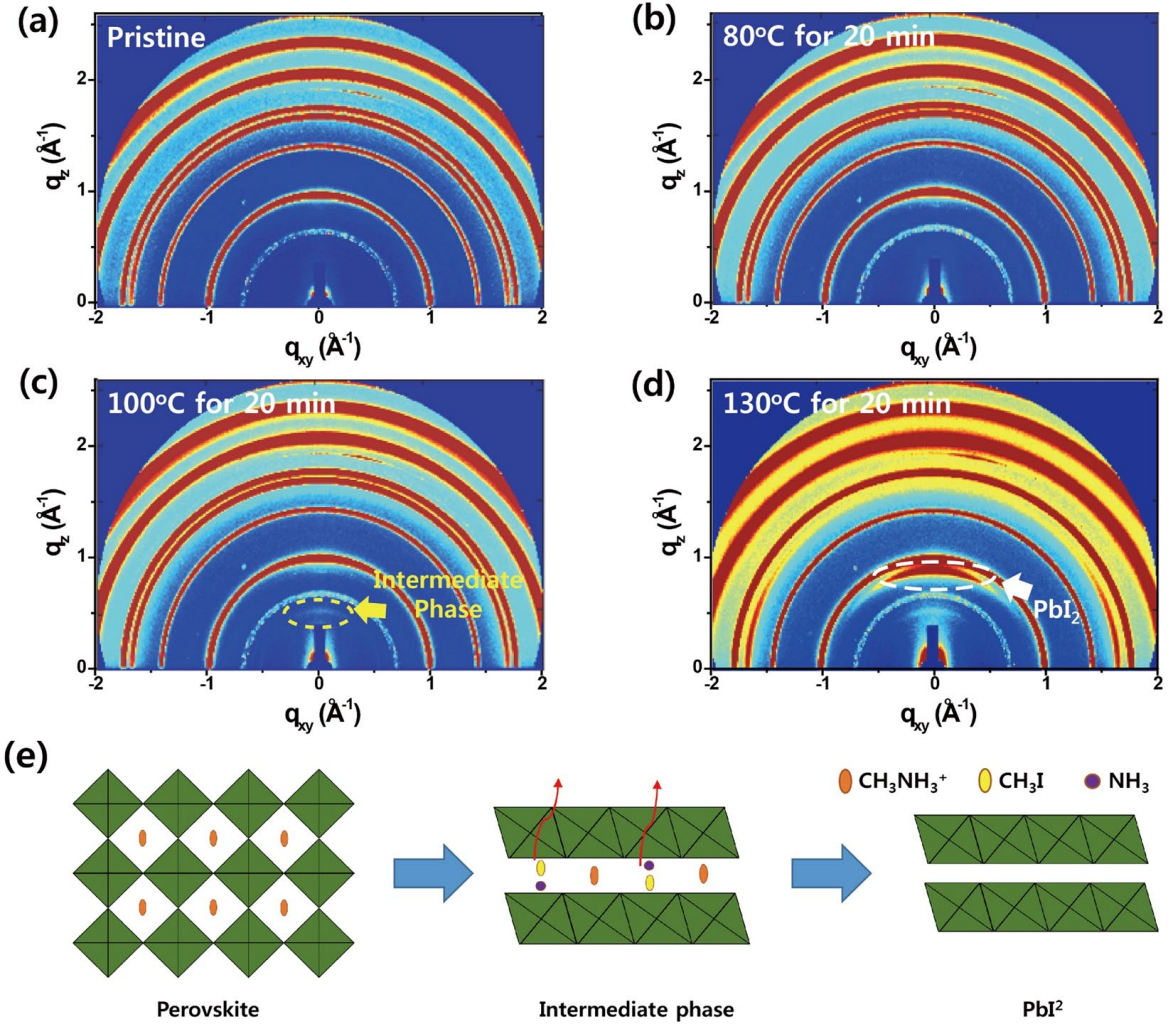
*In-situ* 2D GIWAXD patterns of MAPbI_3_ perovskite films determined under different thermal conditions. (**a**) Pristine MAPbI_3_, MAPbI_3_ exposed to (**b**) 80 °C for 20 min, (**c**) 100 °C for 20 min, and (**d**) 130 °C for 20 min. (**e**) Cartoon representation of the degradation progress in MAPbI_3_ perovskite materials.

As described above, no significant changes were observed in bulk crystallinity, as evidenced by comparison of one-dimensional XRD patterns of encapsulated MAPbI_3_ films before and after the application of heat stress. Therefore, we employed a more sensitive 2D *in-situ* GIWAXD analysis (Pohang Accelerator Laboratory (PAL)) to examine the changes in surface crystallinity under various thermal conditions (Fig. 3a–d). Perovskite film (500 nm) was analysed at an incidence X-ray angle of 0.2 degrees, which diffracted to a depth of 125 nm. The pristine MAPbI_3_ film exhibited planes typical of tetragonal MAPbI_3_ perovskite, which is the crystalline form expected at room temperature^31–33^. The collected 2D GIWAXD images all showed the presence of an MAI peak at q ≈ 0.7 Å^−1^, which can be attributed to the fact that an excess amount of MAI was added during the preparation stage^34, 35^. During the measurements, the prepared MAPbI_3_ perovskite film was either heated and analysed simultaneously or measured after a cycle of heating and cooling to room temperature, performed in the analysis chamber under vacuum conditions. As the heating procedure progressed (over 80 °C), the peak at q ≈ 1.65 Å^−1^ was found to disappear—a finding that is consistent with the appearance of cubic symmetry^36, 37^ (Fig. S2). These peak changes indicate that the tetragonal phase of MAPbI_3_ was transformed into the cubic phase during the heating, and it returned into the tetragonal phase after cooling to room temperature. Although the film heated at 80 °C for 20 min displayed a pattern similar to that of the pristine film, an interesting peak appeared at q ≈ 0.55 Å^−1^ after heating at 100 °C for 20 min (Fig. 3c). Surprisingly, a similar peak near q ≈ 0.55 Å^−1^ was also observed during MAPbI_3_ synthesis process when precursors (MAI and PbI_2_) were converted to MAPbI_3_^33, 34^— this peak was generally associated with the intermediate phase formed by PbI_2_ planes intercalated by MAI (or MA) and solvent because it has longer interplanar distances than the normal PbI_2_ peak. In our case, the peak at q ≈ 0.55 Å^−1^ is expected to be an intermediate phase in which the decomposed molecules are intercalated before being completely degraded to PbI_2_ during the thermal degradation process. We reasonably inferred that the intercalated molecules are thermally dissociated MA or thermally decomposed CH_3_I and NH_3_ from MAPbI_3_ because there was no remained solvent (Fig. 3e). By *in-situ* NEXAFS measurement, we found the behaviour of intercalated MA in the intermediate phase for the first time. This finding will be discussed in the next section. The film heated at 130 °C for 20 min showed a strong peak at q ≈ 0.9 Å^−1^, which was assigned as the (001) plane of trigonal PbI_2_. These results indicate that the tetragonal phase was transformed into the cubic phase during heat stress application, and the onset temperature of thermal degradation was estimated at 100 °C. The cubic phase of MAPbI_3_ perovskite started to decompose while going through the intermediate phase, leaving only PbI_2_ once CH_3_I and NH_3_ evaporated^38^. In addition to the rapid thermal degradation that took place when the sample was heated for a short time at 100 °C, long-term application (1 h) of heat stress also led to thermal decomposition at lower temperature of 80 °C (Fig. S3). After sample exposure to 80 °C for 60 min, the film exhibited the intermediate phase peak at q ≈ 0.55 Å^−1^. After an overall exposure time of 120 min, an additional weak peak arising from PbI_2_ (q ≈ 0.9 Å^−1^) was observed. After cooling to room temperature, the peaks associated with the intermediate phase and PbI_2_ remained, thus demonstrating that the thermal degradation was irreversible despite the removal of heat stress. The thermally degraded film was analysed using 2D GIWAXD at different incident angles (0.1° ≈ 5 nm, 0.15° ≈ 55 nm and 0.2° ≈ 125 nm), and we confirmed that the thermal degradation occurred in the direction from the film surface to the film bulk (Fig. S4). These results indicate that the thermal degradation occurs predominantly on the surface and might, therefore, affect the interface between MAPbI_3_ and the transport layers. Overall, these results explain the significant decrease in the PCE of PeSCs exposed to 85 °C heat stress for several hours, even though no notable changes were observable by the naked eye and the measurement of transmittance and bulk XRD.

**Figure 4:**
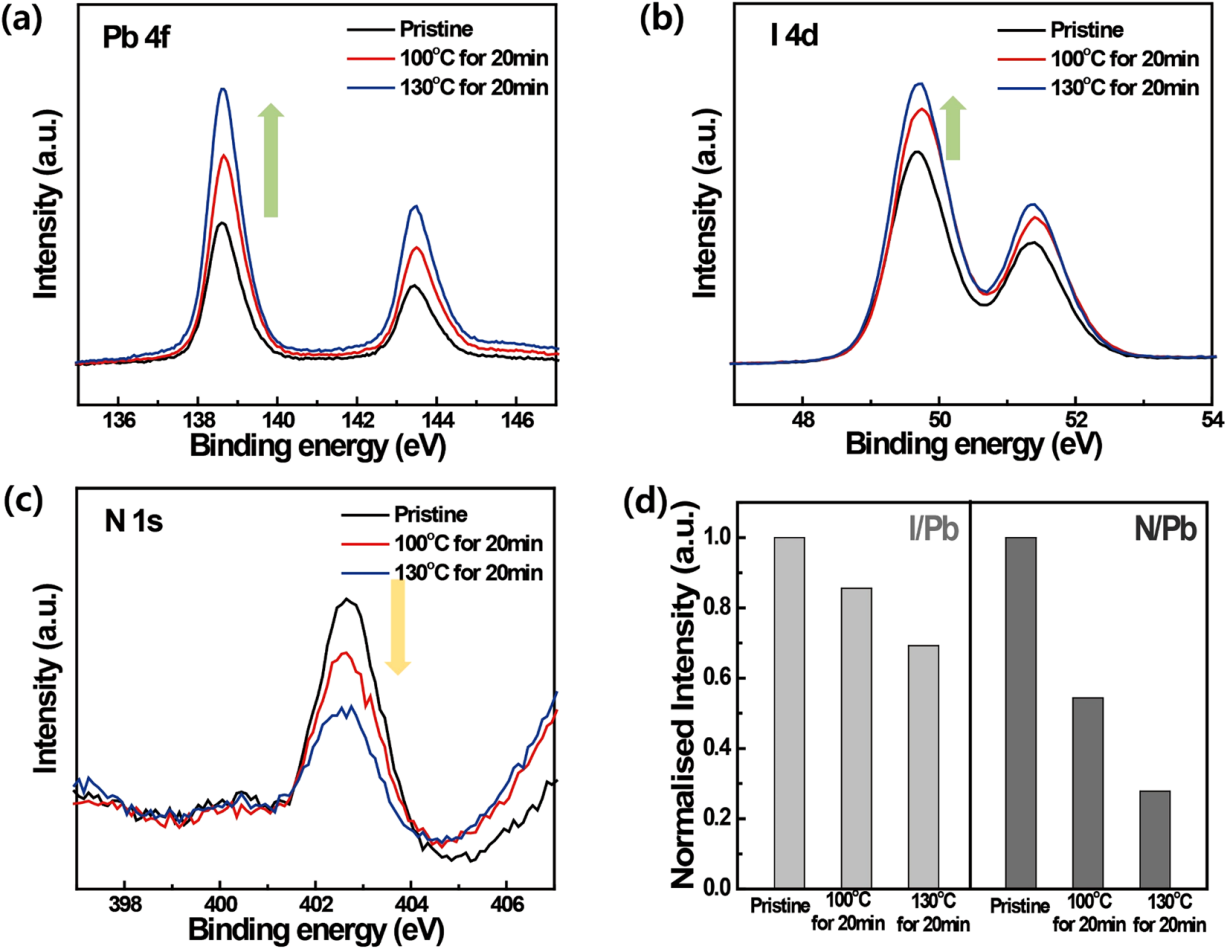
*In-situ* HR-XPS spectra determined under different temperature conditions for (**a**) Pb 4 f (recorded with an excitation energy of 200 eV), (**b**) I 4d (200 eV), and (**c**) N 1 s (500 eV) of MAPbI_3_. (**d**) Changes in the N/Pb and I/Pb ratios as a function of heat exposure.

To confirm the thermal decomposition of the MAPbI_3_ perovskite surface, we utilised *in-situ* HR-XPS to determine the composition ratio, similar to Liu *et al*., who confirmed the growth of MAPbI_3_^39^. *In-situ* HR-XPS measurements were conducted under ultra-high vacuum conditions, as the analysis of MAPbI_3_ components changes depending on different temperatures (Fig. 4). The *in-situ* heating and cooling processes were conducted in the analysis chamber of the spectrometer. HR-XPS is a very surface sensitive technique—in this study, the spectra investigated the top 10 nm of the prepared samples. The results showed that the atomic component ratio of MAPbI_3_ perovskite films changes with temperature. Specifically, the peak arising from Pb 4 f almost doubled in size after heating at 130 °C for 20 min (Fig. 4a), while the peaks associated with I 4d increased only slightly (Fig. 4b) by about 20–30%. Additionally, the peak arising from N 1 s decreased by about ~20–50% compared to that in the pristine sample (Fig. 4c). These atomic composition ratio results indicate that the surface top 10 nm changed to a region rich in Pb and I, but poor in MA. When the peaks arising from Pb, I, and N were normalised based on the intensity of the initial film, N/Pb ratio decreased by 45% and 75%, and I/Pb ratio decreased by 15% and 30% after heating at 100 °C and 130 °C for 20 min, respectively (Fig. 4d). Juarez-Perez *et al*. reported the thermal decomposition of MAPbI_3_ through thermogravimetric analysis and differential thermal analysis (TG-DTA) coupled with quadrupole mass spectrometry (MS), and reported that MAPbI_3_ started to decompose to CH_3_I, NH_3_ and PbI_2_ at 294 °C^38^. These results indicate that the surface of the MAPbI_3_ film decomposed to PbI_2_, CH_3_I, and NH_3_ after being heated at >100 °C, and only PbI_2_ remained on the surface after CH_3_I and NH_3_ evaporated. We assume that the thermal decomposition temperature is significantly lower than that reported in a recent study because of two reasons: 1) we focused on surface degradation and hence could identify even slight changes occurred in the limited region at low temperatures; 2) ultra-high-vacuum conditions can accelerate the degradation of CH_3_NH_3_PbI_3_ even at low temperatures.

**Figure 5:**
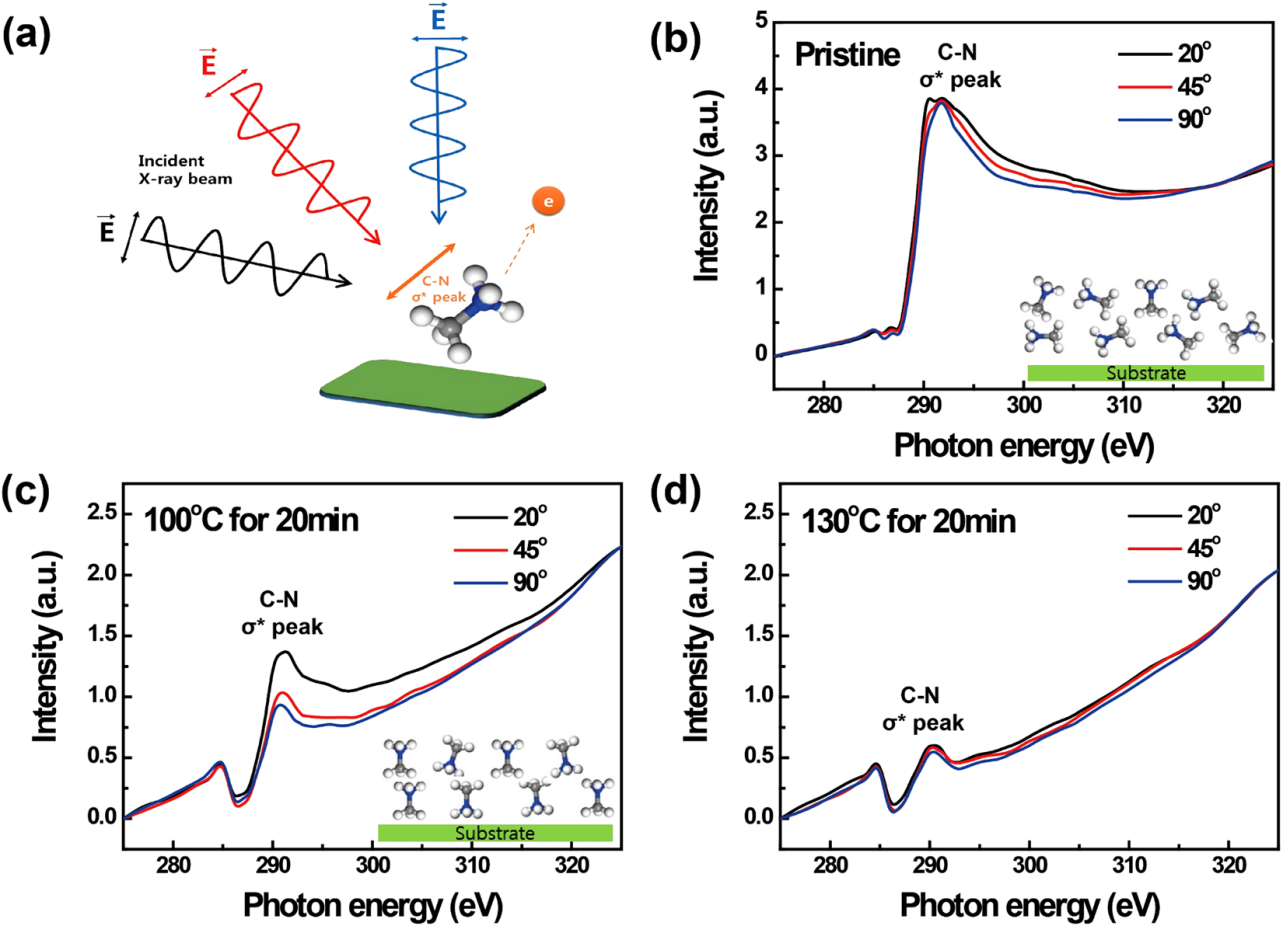
(**a**) Schematic diagram of NEXAFS measurements. Angle resolved carbon K-edge *in-situ* NEXAFS spectroscopy of MAPbI_3_ perovskite film (**b**) before and after heating to (**c**) 100 °C for 20 min, and (**d**) 130 °C for 20 min.

*In-situ* NEXAFS measurements were performed next in order to examine the behaviour of the MA cations, observed in the intermediate phase during the GIWAXD analysis in more detail. The relationship between the molecular orientation of CH_3_NH_3_^+^ cations within the MAPbI_3_ perovskite layer and temperature was probed using *in-situ* NEXAFS measurements under ultra-high vacuum conditions (Fig. 5). Determination of the ensemble-averaged orientation of molecular bonds, such as the C–N bond shown in Fig. 5a, can be accomplished with the acquisition of NEXAFS spectra at different incident X-ray angles due to the linear polarisation of synchrotron X-rays^40, 41^. In the pristine MAPbI_3_ perovskite film, the peak associated with the C–N σ bond (~292 eV) exhibited similar intensity under three different incident angles. However, the peak obtained by grazing incidence X-ray (20°) showed higher intensity than the X-ray utilising higher incidence angles after the sample was heated at 100 °C for 20 min. These results suggest that while the organic CH_3_NH_3_^+^ cations were randomly oriented in the pristine perovskite film, they are oriented in direction orthogonal to that of the substrate after heating. Taking into consideration the results of GIWAXD analysis performed under the same conditions, it can be assumed that the orthogonal orientation of the CH_3_NH_3_^+^ cations is localised to the area between the two PbI_2_ layers prior to the decomposition to CH_3_I and NH_3_ in the intermediate phase. In addition, the peak intensity of the C–N σ bond decreased as the temperature increased, thereby indicating the evaporation of MA molecules—an outcome that is in agreement with the results of HR-XPS analysis. Xu *et al*. reported the dissociation of MA into CH_3_I and NH_3_ defects in a perovskite structure, as observed in the present work^25^. The comprehensive results obtained using a combination of *in-situ* GIWAXD, XPS, and NEXAFS spectroscopy measurements performed at high temperature showed that the orientation and decomposition of MA cations is strongly linked with the progress of thermal degradation in MAPbI_3_ perovskite films. These outcomes suggest that the cationic components of perovskites play an essential role in determining thermal stability.

## Conclusion

In conclusion, we investigated the thermal degradation of MAPbI_3_ perovskite using various *in-situ* surface analysis techniques. The stability of PeSCs in this work was improved using the encapsulation process. Nevertheless, while encapsulation protected the fabricated devices from moisture, it did not provide protection against thermal degradation, even though no significant changes were visible by the naked eye. Through the application of *in-situ* GIXAXS, HR-XPS, and NEXAFS spectroscopy, we were able to systematically study the thermal degradation processes in MAPbI_3_-based perovskite films. MAPbI_3_ in the tetragonal phase was transformed into the cubic phase at high temperatures, and the thermal degradation process started at 100 °C, with the appearance of the intermediate phase. In the pristine material, the C–N σ-bonds were oriented randomly, however, their orientation changed after the application of heat stress (100 °C), adopting instead an orientation orthogonal to that of the substrate. The MAPbI_3_ perovskite decomposed to CH_3_I, NH_3_, and PbI_2_—after the evaporation of CH_3_I and NH_3_, however, only PbI_2_ remained on the surface. These thermal degradation processes occurred progressively from the film surface to its bulk, even when the material was exposed at 80 °C for extended time (>60 min). The results showed that the efficiency of MAPbI_3_-based PeSCs declined significantly at temperatures of ca. 85 °C as a result of the decomposition of MAPbI_3_ and the degradation of the interface between the light-absorber and transport layers. These results provide the important advances in understanding the thermal degradation mechanism of perovskite materials, and suggest that the portion of the perovskite structure incorporating organic cations is very vulnerable to heat stress, and substitutions of MA are needed in order to improve the thermal stability of PeSCs in the future.

## Methods

### Preparation of perovskite solar cells

7 Ω/□ fluorine-doped tin oxide (FTO) glass was employed as the substrate in this study. The substrate was cleaned with acetone, ethanol, and IPA (isopropyl alcohol), followed by 30 min UV ozone treatment. Compact TiO_2_ and mesoporous TiO_2_ layers were fabricated by spin-coating using the respective solutions. For the compact TiO_2_ layer, 0.15 M titanium diisopropoxide bis(acetylacetonate) (75 wt%, Sigma-Aldrich) and 1-butanol (ACS reagent, ≥ 99.4%, Sigma-Aldrich) were mixed and applied as the coating, followed by annealing at 500 °C for 15 min. The mesoporous TiO_2_ layer coating was fabricated using a solution of TiO_2_ paste (18NR-T transparent titania paste, Dyesol), terpineol (mixture of isomers, anhydrous, Sigma-Aldrich), and ethanol (pure, 200 proof, anhydrous, Sigma-Aldrich), mixed in a 1:4:2 ratio (wt%), followed by annealing at 550 °C for 60 min. Following the formation of the mesoporous-TiO_2_ layer, CH_3_NH_3_PbI_3_ perovskite light-absorbing layer was formed using a single step spin coating method by dripping with diethyl ether in a glove box under argon atmosphere^42^. The MAPbI_3_ solution was prepared by dissolving 50 wt% of 1:1:1 MAI (methylammonium iodide, Dyesol), PbI_2_ (99.9985% metal basis, Alfa Aesar), and DMSO (dimethyl sulfoxide, anhydrous, ≥ 99.9%, Sigma-Aldrich) in DMF (*N*,*N*-dimethylformamide, anhydrous, 99.8%, Sigma Aldrich). This solution was spin-coated and annealed at 65 °C for 1 min, followed by 100 °C for 5 min. The hole-transport layer was comprised of Spiro-MeOTAD (2,2ʹ,7,7ʹ-tetrakis(*N*,*N*-di-*p*-methoxyphenyl-amine)-9,9ʹ-spirobifluorene, Lumtech) doped with Li-TSFI (bis(trifluoromethane)sulfonimide, 99.95% trace metals basis, Sigma-Aldrich). The hole transport material (HTM) solution was prepared by mixing Spiro-MeOTAD (72.3 mg), chlorobenzene (1 mL, 99.8% Sigma-Aldrich), 4-tert-butyl pyridine (28.8 µL, 96%, Sigma-Aldrich), and Li-TSFI solution (17.5 μL, 520 mg Li-TSFI in 1 mL of anhydrous acetonitrile (99.8%, Sigma-Aldrich)). Finally, a 100 nm Au electrode was deposited by thermal evaporation. The fabricated perovskite solar cells were encapsulated with cover glass, UV curable edge-sealant for OLED encapsulation, and an adhesive (NOA 88, Norland). Encapsulation procedures were carried out at room temperature under inert atmosphere. Firstly, the sealant was daubed in the marginal glass area of the device and covered with glass, followed by the UV-curing procedure for 30 min at each side. Secondly, the adhesive was smeared between the substrate and the cover glass for robust encapsulation, followed by the UV-curing procedure for 5 min at each side. During the UV-curing procedure, the perovskite area was covered with an aluminium foil to prevent degradation by UV-light. To check the penetration of moisture, water sensitive paper (20301–1 N, TeeJet Technologies) was inserted between the substrate and the cover glass during the encapsulation process.

### Characterisation of perovskite solar cells

The photocurrent J–V performance of perovskite solar cells was assessed using a Keithley 2400 source meter under an AM 1.5 G 1-sun solar simulator (WACOM WXS-155S-10 class AAA). The light source intensity was optically calibrated to one sun (100 mW/cm^2^) against an encapsulated 156 cm × 156 cm sized Si standard solar cell. Current-voltage measurements of encapsulated reference Si solar cell was performed at Korea Institute of Energy Research (KIER). The amount of light was corrected to have a current value with an error rate within ±1% based on the reference solar cell. The J–V curves were obtained using a delay time of 500 ms at each point (26 mV/s) in the reverse scan (RS) from the open-circuit to short-circuit and in the forward scan (FS) from the short-circuit to the open-circuit direction. A defined area of 0.15 cm × 0.5 cm metal mask coated with black non-reflective materials was used for the measurement. The aperture area of the mask was confirmed through a microscope at KIER. The experiments at 25 °C, 85% RH and 85 °C, 85% RH conditions were conducted in a temperature and humidity controllable chamber (Environmental chamber, Woowon Tech, Korea). The experiments at 85 °C, 0% RH conditions were performed in a glove box using a hot plate. The humidity inside the glove box filled with Ar was controlled to a moisture concentration of 0.2 ppm or less through the moisture gauge. (Model AMT, Alpha moisture system, England).

### 2D GIWAXD

2D GIWAXD measurements were conducted at the PLS-II 9 A U-SAXS beamline of Pohang Accelerator Laboratory (PAL) in Korea. The X-rays originating from the in-vacuum undulator (IVU) were monochromated (wavelength λ = 1.068 Å) using a double crystal monochromator and focused both horizontally and vertically (FWHM 300 μm (H) × 30 (V) μm at sample position) using K-B type mirrors. Vacuum GIXD system was equipped with a 7-axis motorised sample stage for the fine alignment of thin films. GI-WAXD patterns were recorded using a 2D CCD detector (Rayonix SX165, USA).

### HR-XPS and NEXAFS

Spectroscopic analysis was carried out at the 4D PES beamline of PAL. Each prepared sample was packed in a vacuum-sealed container and unpacked in the N_2_-overflowing globe tube connected directly to the load-lock chamber. Sample was attached to the Mo heatable holder and loaded into the vacuum chamber without air exposure. The analysis chamber (base pressure: 5 × 10^−10^ Torr) was equipped with an electron analyser (R3000, Scienta) and an X-ray absorption spectroscopic detector adjusted for the observation of the same sample focal point. The acquisition of all spectroscopic data, including the annealing process, was done in an *in-situ* manner.

## Electronic supplementary material

Supplementary information
