# Supplementary material for: Investigation of Thermally Induced Degradation in CH3NH3PbI3 Perovskite Solar Cells using In-situ Synchrotron Radiation Analysis
Source: Sci Rep. 2017 Jul 5;7:4645. doi: 10.1038/s41598-017-04690-w (PMC5498566; doi:10.1038/s41598-017-04690-w)
Supplement: Supplementary file 1 — Supplementary information [file 41598_2017_4690_MOESM1_ESM.pdf]

Supporting Information

Investigation of Thermally Induced Degradation in $CH_3NH_3PbI_3$ Perovskite Solar Cells using *In-situ* Synchrotron Radiation Analysis

Nam-Koo Kim[1,+], Young Hwan Min[1,+], Seokhwan Noh[1], Eunkyung Cho[1], Gitaeg Jeong[1], Minho Joo[1], Seh-Won Ahn[1], Jeong Soo Lee[1], Seongtak Kim[3], Kyuwook Ihm[2], Hyungju Ahn [2], Yoonmook Kang[3], Hae-Seok Lee[3] & Donghwan Kim[3,*]

[1] *Materials & Devices Advanced Research Institute, LG Electronics, Seoul 06763, Republic of Korea*

[2] *Beamline Research Division, Pohang Accelerator Laboratory, Pohang, Kyungbuk 37673, Republic of Korea*

[3] *Department of Materials Science and Engineering, Korea University, Seoul, Republic of Korea*

**Correspondence and requests for materials should be addressed to D.K. (E-mail: donghwan@korea.ac.kr)*

[+]These authors contributed equally to this work

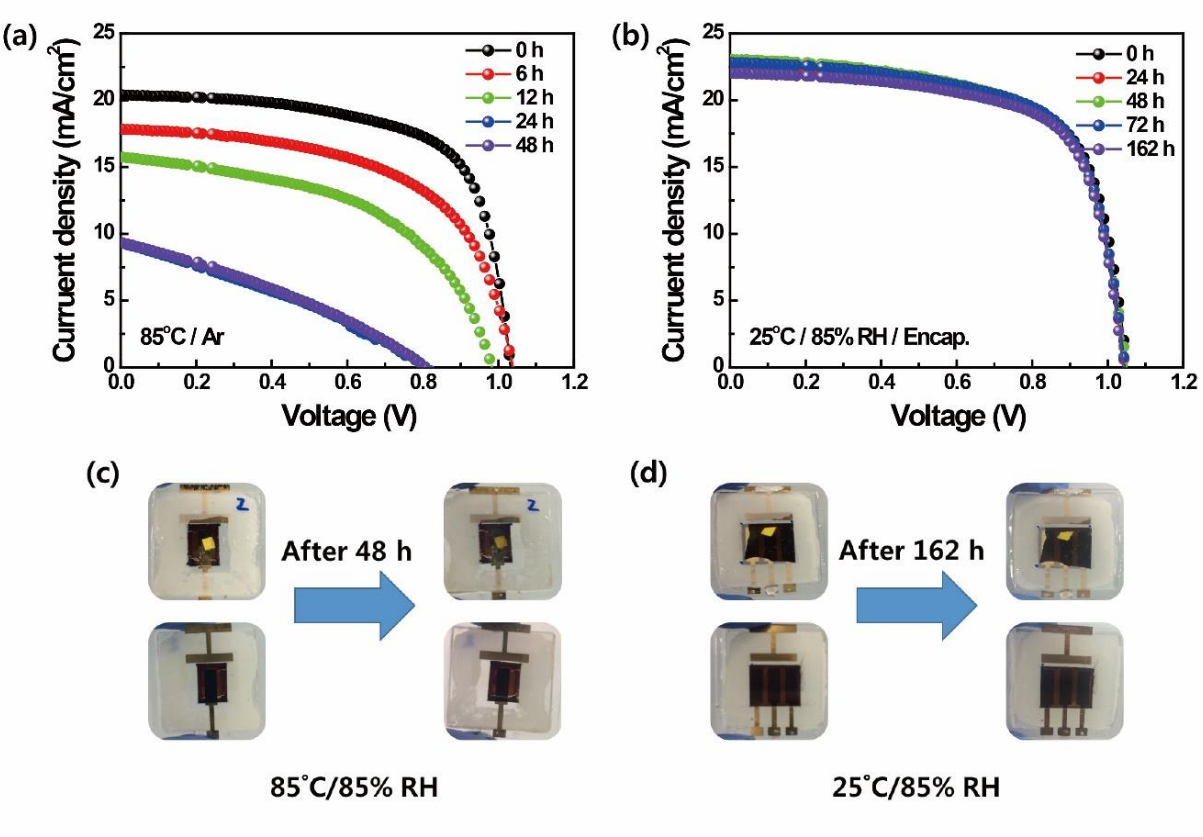

Figure S1. (a) Current–voltage curves determined for $MAPbI_3$-based PeSCs as a function of exposure time at 85 °C/Ar and (b) 25 °C/85% relative humidity (RH)/encapsulation conditions. (c) Colour changes observed in the encapsulated $MAPbI_3$ PeSCs before and after heating to 85 °C/85% RH for 48 h and (d) 25 °C/85% RH for 162 h. Water sensitive paper (yellow) was inserted between the substrate and the cover glass during the encapsulation process.

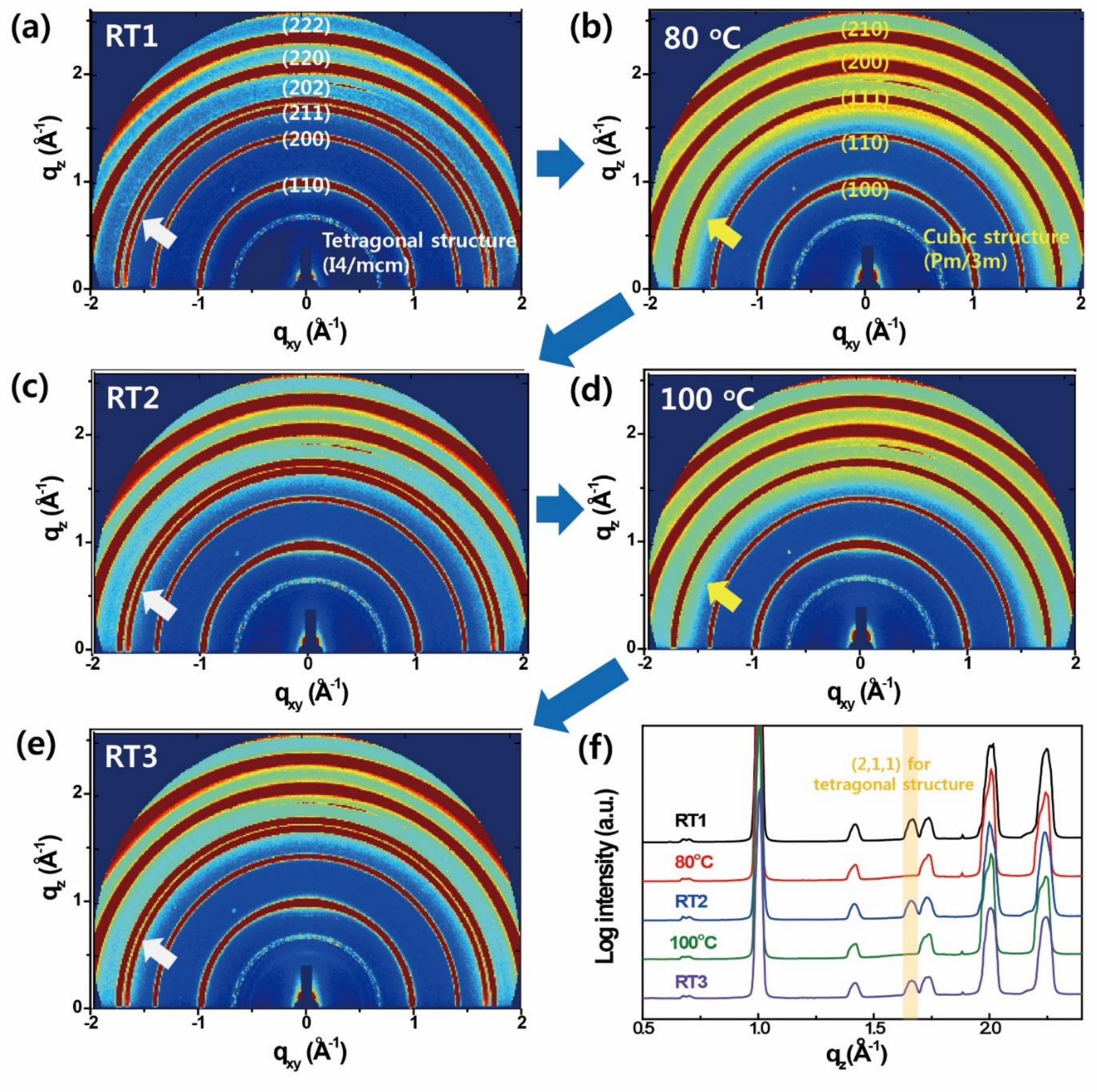

Figure S2. *In-situ* 2D GIXRD patterns of $MAPbI_3$ perovskite films exposed to different thermal conditions. (a) Pristine films at room temperature (RT1) and films (b) examined during the exposure to 80 °C heating, (c) after cooling to room temperature (RT2), (d) during exposure to 100 °C, and (e) after cooling to room temperature (RT3). (f) Out-of-plane GIWAXD line profiles determined under different conditions.

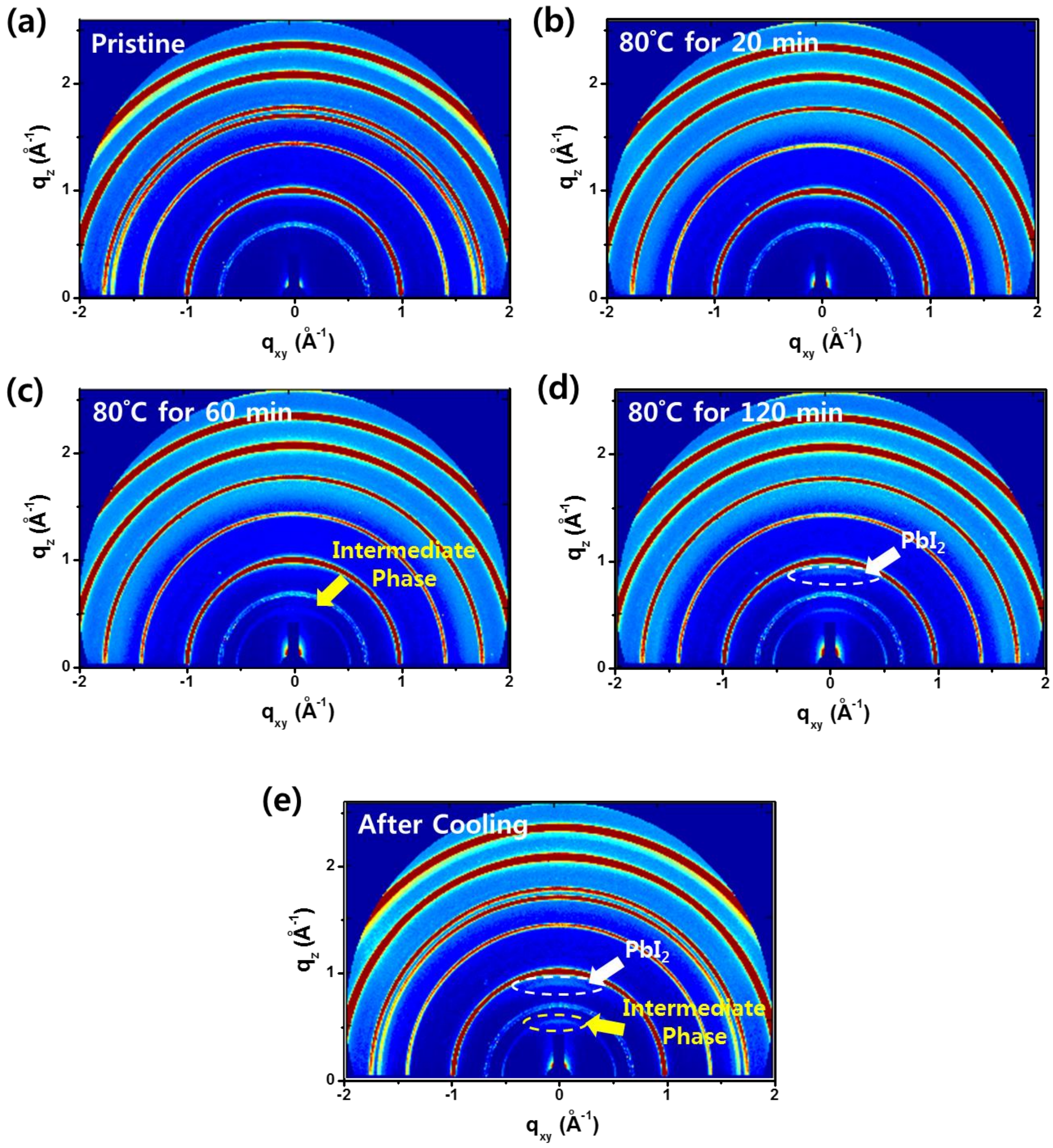

Figure S3. *In-situ* 2D GIXRD patterns of $MAPbI_3$ perovskite films exposed to 80 °C heat stress. (a) Pristine films. Films exposed to heating for (b) 20 min, (c) 60 min, (d) 120 min, and (e) after cooling to room temperature.

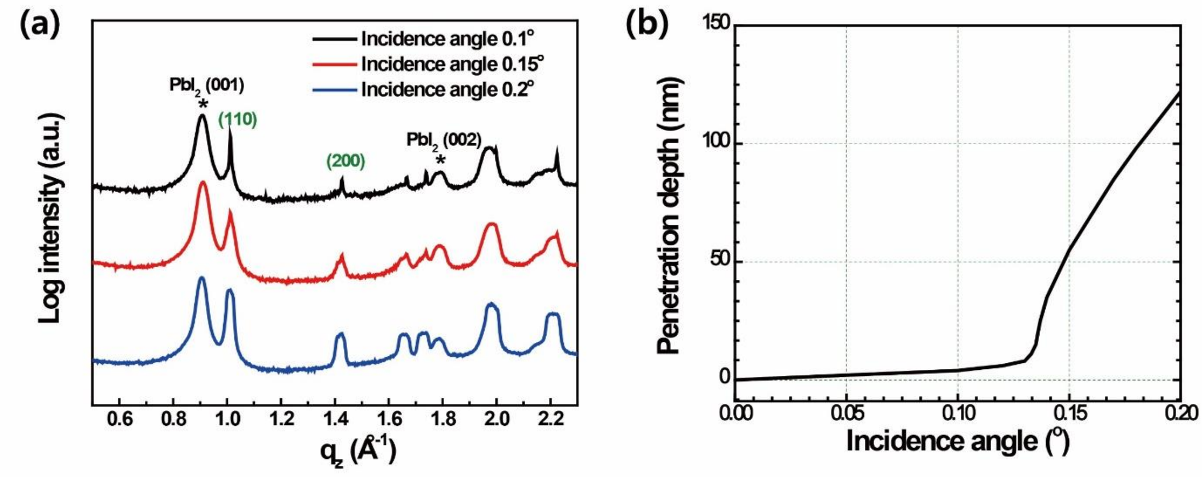

Figure S4. (a) Out-of-plane GIWAXD line profiles of a thermally degraded $MAPbI_3$ perovskite films measured at different incidence angles. (b) Incidence angle *versus* attenuation length determined for $MAPbI_3$ perovskite films. ($MAPbI_3$ density = 2.2, Energy = 11600 eV)

Table S1. Penetration depth and PbI_2 (001)/$MAPbI_2$ (110) ratio as a function of X-ray incidence angle.

	**Incidence angle 0.1˚**	**Incidence angle 0.15˚**	**Incidence angle 0.2˚**
**Penetration depth (nm)**	5	55	125
**Ratio PbI_2 (001)/$MAPbI_2$ (110)**	16.1	9.8	2.7
